# Dental Implants in Posterior Maxilla: Diagnostic and Treatment Aspects

**DOI:** 10.1155/2012/132569

**Published:** 2012-09-13

**Authors:** Dogan Dolanmaz, Figen Cizmeci Senel, Zafer Özgür Pektas

**Affiliations:** ^1^Department of Oral and Maxillofacial Surgery, Faculty of Dentistry, Selcuk University, Konya, Turkey; ^2^Department of Oral and Maxillofacial Surgery, Faculty of Dentistry, Karadeniz Technical University, 61080 Trabzon, Turkey; ^3^Department of Oral and Maxillofacial Surgery, Faculty of Dentistry, Baskent University, 06490 Ankara, Turkey

Dental implantology is one of the most popular and intensively researched topics of current dental medicine. The necessity for the former complicated preprosthetic surgical procedures to facilitate the partial dentures has recently been decreased with the widespread construction of implant-supported prosthesis. Nevertheless, the alveolar deficiency that impedes the insertion of dental implants makes a number of similar reconstructive procedures inevitable. The posterior maxilla is one of the most challenging anatomic locations for the implant placement that requires adjunctive surgical procedures. This special issue covers leading researches and reviews on this topic that we believe would contribute to clinicians. 

Insufficient bone quality and quantity in posterior maxilla is a common clinical state which makes the implant applications challenging in this site. The main reason for that is the pneumatization of sinus subsequent to the tooth loss and the concomitant excessive alveolar resorption. Insufficient subantral bone and coexisting increased interarch distance necessitate a combined sinus lifting and augmentation procedures. T. Kanno et al. reported the results of their retrospective study entitled *“*Simultaneous sinus lifting and alveolar distraction of a severely atrophic posterior maxilla for oral rehabilitation with dental implants*”* in which they performed combined sinus lifting and alveolar distraction osteogenesis in a case series with 27 individuals. The study revealed a histomorphometric similarity between the new bone formed by the presented technique and the bone generated via the sinus lifting only. They also accomplished stable implant rehabilitations within this topic.

The quality and the quantity of the host bone are the key determinants for successful dental implants. The quantity of the bone may be estimated via advanced radiological techniques in a high precision. However, a definitive accuracy cannot be pronounced although a number of techniques are available to evaluate the quality of bone. H. Bilhan et al. have reviewed the current techniques used for the estimation of the quality of the host bone while they simultaneously reported the results of a pilot experimental study that compares the densitometric evaluation of the dental volumetric tomography (DVT) with micro CT with their study entitled *“*How precise is dental volumetric tomography in the prediction of bone density?*”.* The results of this study revealed that the Hounsfield unit evaluation via DVT is not a reliable method to evaluate the density of bone.

C. Riben and A. Thor have published their review on “graftless augmentation procedures” which became a popular topic on sinus floor elevation, regarding a conventional surgical approach for the posterior maxillae. Their study entitled *“*The maxillary sinus membrane elevation procedure: augmentation of bone around dental implants without grafts—a review of a surgical technique*” *includes the technical details of the procedure.

The alveolar bone loss principally initiates with the tooth loss and this state may complicate the ideal placement of the implants. A number of surgical methods is advocated to prevent the bone loss. G. Pagni et al. reviewed these techniques in their study entitled *“*Postextraction alveolar ridge preservation in the molar area: biological basis and treatments*”. *The study provides comprehensive information about the socket healing and biology of the alveoloar bone resorption subsequent to extraction. They stated that the improvements in the grafting technologies would give rise to less invasive surgical interventions.

The recent advances in the implant surface characteristics led to major modifications in former fundamentals. An increased osseointegration rate with the improvement of the surface characteristics of dental implants resulted in a numerous successful reports for the implants shorter than 10 mm. Today, many companies appear in the dental market with their recently introduced 6 mm or shorter dental implants. Owing to this, clinicians now can be able to offer effective and noninvasive remedies to their patients avoiding advanced complicated surgeries in case of severe alveolar atrophy. Although a number of clinical reports reveals high success rates for the mandible, the use of short implants in the maxilla is still debatable especially for the single-tooth replacements due to its porous nature. D. Lops et al. compared the clinical success rates for 8 mm. Implants with that of 10 mm. In their long-term study entitled *“*Short implants in partially edentulous maxillae and mandibles: a 10 to 20 years retrospective evaluation*”.* In general, they declared similar success rates for 8 mm and longer implants. The results of this study revealed that the use of 8 mm implants seems to be safe in the posterior maxilla.

